# The Arsenical Treatment of Tooth-Pulps

**Published:** 1886-02

**Authors:** A. Morsman

**Affiliations:** Iowa City.


ARTICLE III.

### THE ARSENICAL TREATMENT OF TOOTH PULPS.

BY A. MORSMAN, M. D., D. D. S., IOWA CITY.

It is with considerable hesitancy that I approach the subject of pulp devitalization. There are many among you older in practice than I, to whom my words will come with the monotone of an oft-repeated story. It must be remembered, however, that error does not give way to a single assertion, but to repetition and reiteration of truths.

As long ago as 1868, Prof. Flagg inveighed against the accepted doctrines regarding the use of arsenic, but nothing seems to have come of it, and teachers and writers go on promulgating the same vague and timidity-inspiring ideas, throwing students into the profession to learn by long and tedious experience, how to use this remedy to confer the most good and the least suffering upon their patients. In looking over standard text-books, I am impressed with the belief that he who begins practice with no more knowledge of this operation than he can obtain from this source, will count his failures upon his fingers, and his successes upon his thumbs.

The preparation of root canals for filling, from its inception to its termination, is an exceedingly difficult thing to do well, and an operation requiring great delicacy of manipulation to accomplish the object sought with the least infliction of suffering. This latter consideration is of the greatest importance in attempting devitalization.

Pain is an element that enters largely into almost all our operations, and to those whose dental imperfections *should* induce them to seek our aid, it is an obstacle, and one that is increased by every pang that we inflict. It is an obstacle to our success, also, by presenting to us, instead of simple cavities, easily managed and giving the promise of great utility, complicated cases, delay induced requiring great skill, and promising a lesser reward for our labors.

Commercial arsenic, or arsenious acid, has been used since 1836 for devitalizing tooth pulps. It has been misused and abused, but for all that it has become the accepted means to this end, and the number of pulps destroyed in other ways is proportionately very small.

Arsenic is a caustic irritant, and as such acts upon the dental pulp—just how, I do not pretend to say, but I see no reason for assuming that it is very different from other caustics in mode of action, save as increased potency is a difference. None of the "fine spun" theories that have been advanced throw any light upon the subject, because none of them agree with known facts. One ingenious theory is that the arsenic, being an irritant, causes congestion, congestion strangulation, and the pulp sloughs at the apical foramen. That is a good theory when the facts bear it out, but they don't very often, because only a small portion of pulps die in that way; most of them are obstinate enough to die piece-meal. There is a great temptation to bridge a chasm of ignorance with a theory. One fact is worth a dozen theories, and there are times when it is creditable to say "I don't know."

The preliminary treatment of pulps that are to be subjected to arsenical devitalization demands earnest consideration. An application of arsenic to a tooth pulp in normal condition, if it has been properly made, will produce no pain, and will act with desirable promptitude, but just as there is is deviation from normality in the pulp, so is deviation from comfort to the patient, and the action of the medicament is delayed. I wish to speak impressively, therefore, when I say arsenic should never be applied to a pulp that is congested, or inflamed, or that has ached during the preceding forty eight hours, without preliminary treatment. Ofcourse I do not mean the slight temporary ache that is produced by cold air or drinks Indeed, that is to be looked upon rather as an indication of health than otherwise. Toothache, when the pulp is living, is indicative, usually, of inflammation in one of its stages, of that organ, and this condiion is greatly aggravated by arsenical application. Time and comfort are both gained by first relieving the present symptoms. If only the stage of congestion has been reached, a gentle stream of water thrown against the sides of the cavity will often be sufficient to relieve the pain, and a cotton stopping that will protect from atmospheric changes may be all that is necessary as an application. Filling the cavity with chalk or soda is often an effective remedy. *Usually*, more than this is required, especially if there have been nocturnal pains. We have a number of remedies to choose from —carbolic acid, creosote, oil cloves, oil cinnamon, oil peppermint, menthol, hydrate of chloral, chloroform, opium or its preparations, camphor, and others that I do not remember. I have never been so uniformly successful with anything as with carbolic acid, either full strength or diluted with one of the oils. Whatever remedy is used, it should be allowed to remain in the tooth for twenty-four hours, and, to be effective, must render the tooth painless during that time. If pulpitis exist, it must still be our aim to relieve pain, and to protect from thermal changes and pressure, but this is now much more difficult than in the preceding stages, and we may run the gamut of remedies before we accomplish our purpose. Aconite and morphia are standard remedies here, and the two can be combined with most excellent results. Carbolic acid, creosote, and remedies of that class, are sometimes used with good effect, but it seems to me they are quite as frequently irritants. Rest and time are essential. Applications should not be changed oftener than every other day, unless there are exacerbations of severe pain.

Pulp ulcerations should be treated in the same manner, save that the stopping must not prevent the escape of pus. This is an unnecessary warning when cotton is used, but it is not so as to other temporary stoppings.

It should be borne in mind that a considerable degree of abnormality *may* exist without pain, and we are then dependent upon physical signs in making our diagnosis.

The usual methods of applying arsenic are as a paste and powder. The paste consists of arsenic, creosote, and morphine. The powder is the commercial arsenic. The paste is the most popular form, and I am at a lost to determin why, unless it is the too common reason that because the bell-wether jumped the fence, the other sheep followed. It is sticky, and requires considerable dexterity to place accurately where desired. The morphine I believe to be an entirely useless ingredient, because the arsenic and creosote act first, closing the absorbents so effectually that no morphine can be taken up. What surgeon desirous of introducing a remedy by absorption would first cauterize the surface? The creosote acts as a pain obtruder, but is no more effective than oil of cloves, and is much more unpleasant. I do not like creosote. In my hands it is not nearly so effective as a pain obtruder as carbolic acid, and is about on a par with the essential oils. I speak, of course, of *pure* wood creosote. The commercial article is fully half carbolic acid, and is therefore just that much better than a pure article. A solution of carbolic acid in oil of cloves, or cinnamon, makes a very effective and not unpleasant remedy. It should be almost equal parts. If an application as mild as creosote is desired, I would prefer oil of cloves as being more acceptable and equally efficient. The profession has unfortunately fallen in the habit of creosoting everything in a way that is decidedly empirical. In using the paste, a small portion is taken upon the point of an instrument, or carried to the cavity upon a few fibres of cotton. The powder is used by moistening slightly a very small pellet of cotton in the obtunding solution, touching it lightly to the dry arsenic, and placing it in the cavity with plyers. There is usually enough arsenic adheres to the cork of the bottle for several applications. Only a very small portion of the drug is required, (about one-twentieth grain,) and we use, as a matter of convenience simply, much more than is really necessary, and enough at times to be hurtful.

In regard to preparing a cavity for an arsenical application, I should make it a rule never to give pain. We should be satisfied to syringe with lukewarm water, and to lightly detach loose pieces of debris, leaving the excavating to be done when it can be done without pain. The walls of the cavity should never be broken down except when necessary to gain access.

Having now placed the arsenic in position, our next care is to keep it there. In a cavity with four walls, neither of which impinges upon the gum, this is a very simple matter, bearing in mind, however, that the material used as a stopping must, while it keeps the medicine in the cavity, be sufficiently porous to permit the escape of gases formed by the decomposition of the pulp. This is essential, because patients often fail to keep appointments, and we are obliged to guard against their negligence as well as against incidental danger. Periostitis from decomposing pulp is a serious complication both to patient and operator. There need be no effort made to keep the saliva out of the cavity; the arsenic is but sparingly soluble, and its mechanical retention is all that is necessary. For this class of cavities, a cotton plug is the most efficient means we have. It can be moistened with sandarach if desired, but I do not consider it necessary, and rarely use it. All other cavities than these I have just mentioned require greater care to properly retain the medicament. It will be readily recognized that if the arsenic escapes from a cavity, the margin of which impinges upon the gum, death of the tissue just in proportion to its invasion will follow, and to prevent this, especially in proximal cavities, is often very difficult. Adhering still to cotton as the best plug when applicable, it must be accurately adjusted while the cavity is *dry*, and while still in this condition saturated with a solution of sandarach or shellac. If the walls of the cavity are so badly broken down as to make the retention of the plug unlikely, and ligatures cannot be used to bind it in place, we are obliged to resort to other means, and oxychloride of zinc, gutta-percha, or zinc phosphate, can be used. But it must be remembered that they lack the element of porosity, and therefore need earlier removal. The two latter have another objectionable feature; it is exceedingly difficult to apply them without producing pressure upon the pulp, and pain as a concomitant. *Any application that produces pressure upon the pulp as evidenced by pain is faulty, and should be removed at once.* Attention to this will save your patients nights of agony. The oxychloride acts very nicely in such cases, and can be made to adhere to an almost flat surface if dry. Its use occasions some pain (momentary), which can be decreased by mixing it pretty stiff.

It becomes occasionally necessary to destroy a pulp that is not exposed by the cavity; in such a case, make the application as usual, but allow it to remain only twenty-four hours, when the pulp can be approached with little or no pain, and the arsenic applied directly.

Having now placed the arsenic in position, and taken proper precautions for its retention in the cavity, how long shall we allow it to remain? The teachings, both by text and precept, still continue to be that it is dangerons to permit arsenic to remain in a cavity longer than twenty-four, or at most, forty-eight hours. It is strange that this opinion still persists, in spite of the many opportunities every practitioner has had to demonstrate its falsity. Hundreds of times I have made appointments, which for some reason have been broken, and the arsenic, which I designed to remove at that sitting, has remained in the cavity for weeks and months without harm. Others relate similar experiences. The following case occurred when I first commenced practice, and will serve as an illustration of the folly of such teaching:

I made an arsenical application for a lady patient, and appointed a time for her return. She didn't appear. The next day passed—uneasiness on my part—another and still another day, and no opportunity to remove what had now become a night-mare to me. The fifth day I could stand it no longer, and slipping a pair of forceps into my pocket, I went to the lady's residence, expecting nothing *less* than that my patient was in bed with a face swollen all out of shape. I was greatly relieved when she met me at the door. She had had company—couldn't come—would be down to my office that afternoon. That day she left town. *Three months* afterwards I removed that application, cleaned out the canals, filled the tooth, and all was well. She said she never had a tooth filled so easily in her life. It was a case of vicarious sacrifice. I was the fellow who suffered.

We have had several cases of deaths *ascribed* to arsenic used as a pulp devitalizer. One occurred not long since. How does this happen? Easy enough. The attending physician, unable, from obscurity or ignorance, to make a correct diagnosis of the case, charges it upon the arsenic, and the dentist who put the plug in the mouth, *who ought to know better*, and whose ignorance is directly chargeable to his preceptor, or Alma Mater, permits the charge to go unchallenged. Suppose, in a case of this kind, a suit for malpractice was brought on the ground that the arsenic had been left in the cavity a week or ten days. (We all do that occasionally, in spite of the books.) What chance of acquittal would the defendant have, if the teachings and opinions of the profession were cited to the average juryman? In self-defense we ought to demand a change.

The dangers from causing the arsenic to remain in a cavity longer than the prescribed time are said to be, first, poisoning from absorption of the drug: second, periostitis, necrosis, etc., from its action through the apical foramen. Arsenic used as we use it *cannot be absorbed.* Acting primarily and immediately as a caustic, it destroys as far as it reaches, and absorption is impossible. It has been used for centuries in the form of Come's powder for cancer, and in the hands of quacks has been applied to every available portion of the human anatomy, and large growths eaten out by it. If arsenic be diluted or reduced by admixture with other substances until it no longer acts as an escharotic, or but weakly so, absorption is imminent; but I believe it to be without danger as long as it is destructive. Dr. Ringer says: "The absorption of arsenic can be effectually prevented if sufficient be employed to excite active inflammation, for inflamed tissue loses the power of absorption more or less completely. * * * If, through fear of poisoning too weak an application is employed, the most certain way is adopted of accomplishing what it is desired to avoid." Our arsenical preparations should, therefore, be at least one-sixth arsenic. The nerve paste, as it is commonly used, is one-fifth arsenic. Come's powder is one-half arsenic.

In answering the first charge I have partly answered the second. Considering the drug to be placed in a root canal where there is no pulp, it might pass through the foramen, and when there, would, of course, act upon the adjacent tissues; but if there is any portion of living pulp in the canal, and that is the only condition requiring its use, then it is effectually prevented from passing through, unless, in very unskilful hands, it is forced through.

The idea of danger being now set aside, we again ask; How long shall the first arsenical application remain in position to produce the best results? It is possible that the arsenic has spent itself in twenty-four hours, but my success seems greater when I prescribe a longer time. Whether that is due to a continued action of the drug, or is simply the effect of time, I am still in doubt, but evidence seems to show that the arsenic does not lose its efficacy at once. It is likely that the breaking down of tissue permits the action of particles not already satisfied. Be that as it may, my practice is to leave the arsenic in position from one day to four *weeks*, according to the convenience of the patient, and the size, age, and temperament of the pulp, *governed, of course, by the considerations previously mentioned in this paper.* Expecting, if the latter time is allowed, that the pulp will have sloughed, and that it can be removed at the next sitting. Preferably, however, if my patient is entirely under my control, and I can be certain of the security of the application, I would allow one week to elapse before attempting any removal of pulp substance. If security of application is an uncertainty, and it is quite often so in proximal cavities, I would make a *preliminary* application for twenty-four hours, to enable me to remove sufficient debris, or pulp, if need be, to make the cavity retaining, and then allow nearly a week to intervene before the next sitting, when I would prepare the cavity, and cut out the bulbous portion of the pulp with a rose bur in the engine, touching lightly, however, that the pressure might not be communicated through the dead portion to that which was capable of giving pain. At this stage the pulp *may* be entirely dead, but my experience is, that such is not often the case, and I would deprecate any attempt at extirpation unless further time was impossible, or evidence of sloughing was conclusive. It is these premature attempts to remove pulps that have given our patients such a horror of this operation.

And now, the bulbous portion being removed, shall we make a second application of arsenic ? In many cases it is not The pulp will slough off anyhow. But we cannot foresee this result, and knowing that these fragments are sometimes very tenacious of life, it is better to make another application at once. And here again I should use the dry arsenic. Tannin in combination has been recommended, on the ground that it hardens the fibrils, and renders them easy of removal. I do not consider hardness an advantage, and would much prefer that the remaining tissue would disintegrate, as it can then be easily wiped out with cotton on a broach. The erroneous belief that tissue devitalized by arsenic would not disintegrate, has been held. While arsenic is one of the most potent preservers of dead animal tissue, that which it destroys does not differ in this respect from that destroyed by any other escharotic.

How long, now, shall this second application remain? It is impossible to say just when the sloughing will occur—the conditions of age, temperament, vitality, etc., are so different. Young pulps are more easily affected than those that are older. The lower the patient stands in the scale of temperaments, the less vitality there is in these fragments, or fibrils, and the sooner they are thrown off.

Here the operator must use his judgment, but I see no reason why the application may not remain *in situ* until sloughing has taken place. There is now no difficulty whatever as to its retention, and the quantity can be even smaller than before. And now I would recommend moistening a fine instrument—dipping it into the dry powder, and thus carrying it directly into the root canals. In inaccessible cavities this is impossible, and resort must be again had to the pellet of cotton, unless it is determined to make an accessible entrance to the pulp cavity, which is by far the best plan. The cavity proper can then be filled at once, and further operations conducted through the artificial opening.

I should not expect sloughing to take place in the average case in less than three weeks from date of first application, and should make an appointment at that time, or as nearly as might be convenient. In the majority of cases, extirpation can now be performed painlessly. If this cannot be done, and manipulation causes suffering, more time is required, and possibly more arsenic, although this is very rarely necessary where the drug has been kept in position the time specified. I usually fill the canals with carbolic acid, and the cavity with loose cotton, permitting the patient to change it when desired, postponing the operation for such time as may seem best, but counting rather in weeks than in days, for nature always works slowly. In diagnosing, life still existing in the root filaments, we must be on our guard against deception by spurious pulp pains. This is caused by pressing the dead pulp, debris, or anything that is in the canal, against the unhealed continuation of the nerve exterior to the apex of the root. To differentiate, use a very fine probe—the finer the better—with a sharp point, and introduce it slowly and carefully along the side of the canal. If the pulp is dead, it will go to the apex without producing pain.

Tapping, as a means of ascertaining the condition of the periosteum, is delusive when a tooth has been under arsenical treatment. All dying pulps give pain upon tapping the tooth. So do those undergoing the later inflammatory stages. A symptom due most likely from periostitis from continuity. It is nothing of moment, and needs no treatment. One noticeable point about this symptom in devitalizing is, that it occurs only at the last stages—may it not be in a measure indicative of sloughing?

I have now only to say a few words about partly formed and temporary teeth. The above practice must be considerably modified in both cases. The same conditions exist in both of these classes of teeth, namely, enlarged apical foramen. We must be on our guard, therefore, against greater ease of forcing the medicament through.

The rule to save all pulps alive, whenever possible is never so pertinent as in the treatment of these teeth, because in permanent teeth partly formed, further growth ceases on death of pulp, that being pulp function. The root of pulpless temporary teeth cannot be absorbed, and may create disfigurement.

It is not necessary to allow so much time in these cases for arsenical action. In temporary teeth, twenty-four hours is long enough, and one application likely to be sufficient. For young children, I would prefer to use frequent applications of carbolic acid or tincture of iron.—*Southern Dental Journal.*

